# Wet‐Spinning Carbon Nanotube/Shape Memory Polymer Composite Fibers with High Actuation Stress and Predesigned Shape Change

**DOI:** 10.1002/advs.202404913

**Published:** 2024-08-09

**Authors:** Meng Li, Kun Chen, Ding Zhang, Ziming Ye, Zifan Yang, Qi Wang, Zhifan Jiang, Yingjiu Zhang, Yuanyuan Shang, Anyuan Cao

**Affiliations:** ^1^ Key Laboratory of Material Physics Ministry of Education School of Physics and Microelectronics Zhengzhou University Zhengzhou 450052 China; ^2^ School of Materials Science and Engineering Peking University Beijing 100871 China; ^3^ Beijing National Laboratory for Molecular Sciences Key Laboratory of Polymer Chemistry and Physics of Ministry of Education Center for Soft Matter Science and Engineering College of Chemistry and Molecular Engineering Peking University Beijing 100871 P. R. China

**Keywords:** actuator, carbon nanotubes, composite fiber, polyurea, spatial deformation

## Abstract

Actuators based on shape memory polymers and composites incorporating nanomaterial additives have been extensively studied; achieving both high output stress and precise shape change by low‐cost, scalable methods is a long‐term‐desired yet challenging task. Here, conventional polymers (polyurea) and carbon nanotube (CNT) fillers are combined to fabricate reinforced composite fibers with exceptional actuation performance, by a wet‐spinning method amenable for continuous production. It is found that a thermal‐induced shrinkage step could obtain densified strong fibers, and the presence of CNTs effectively promotes the tensile orientation of polymer molecular chains, leading to much improved mechanical properties. Consequently, the CNT/ polyurea composite fibers exhibit stresses as high as 33 MPa within 0.36 s during thermal actuation, and stresses up to 22 MPa upon electrical stimulation enabled by the built‐in conductive CNT networks. Utilizing the flexible thin fibers, various shape change behavior are also demonstrated including the conversion between different structures/curvatures, and recovery of predefined simple patterns. This high‐performance composite fibers, capable of both thermal and electrical actuation and produced by low‐cost materials and fabrication process, may find many potential applications in wearable devices, robotics, and biomedical areas.

## Graphical abstract

By combining shape memory polymer (polyurea) and carbon nanotube (CNT) fillers through wet spinning, a thermo/electrically dual‐responsive nanocomposite fiber actuator is fabricated. This composite fiber exhibits excellent actuation performance, including high output stress and rapid response, surpassing most conventional shape memory polymer (SMP)‐based composites. It also possesses programmable spatial deformation characteristics, including shape transformations between 1D, 2D, and 3D.

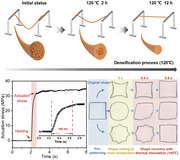

## 1 Introduction

Shape memory polymers (SMP) have attracted much attention as one of the candidates for smart actuators owing to their response to various external stimuli, which have many applications including medical devices,^[^ ^1^ ^]^ soft robotics,^[^ ^2^ ^]^ wearable devices,^[^ ^3^ ^]^ aerospace self‐deploying structures,^[^ ^4^ ^]^ etc.^[^ ^5^ ^]^ As a typical conventional SMP and a thermoplastic elastomer, polyurethane materials usually exhibit large actuation deformation and possess designable on‐demand molecular structures, but the actuation stress (stress output from the material during actuation) remains at low levels and needs to be improved substantially.^[^ ^6^ ^]^ To this end, recently researchers have adopted two main approaches by designing and tailoring the molecular structure of polyurethane,^[^ ^7^ ^]^ or by introducing micro/nanoscale fillers such as carbon nanotubes (CNTs),^[^ ^8^ ^]^ graphene,^[^ ^9^ ^]^ carbon black,^[^ ^10^ ^]^ metal,^[^ ^11^ ^]^ etc.^[^ ^12^ ^]^

For instance, Vaia et al. dispersed 1–5 vol% CNTs into polyurethanes and casted a bulk form nanocomposite that could release an actuation stress of 1.4 MPa under infrared irradiation, 50% higher than that of the original polymer (0.6 MPa).^[^ ^13^ ^]^ Moghim et al. further investigated the thermomechanical properties and shape memory behavior of CNT‐reinforced polyurethane nanocomposites by the same solution casting method and found that the actuation stress was enhanced by about 100% (reaching 2.4 MPa).^[^ ^14^ ^]^ It indicates that CNTs could enhance the mechanical and actuation properties of polyurethanes, but the performance of the resulting nanocomposites was also limited by the existence of defects such as the interfacial cracks between PUs and CNTs as observed in those studies. There have been increasing efforts in recent years in developing SMP‐based nanocomposite actuators in the form of thin film or fibers made by wet‐spinning and 3D printing methods, which showed diverse shape change behavior by structural design.^[^ ^15^ ^]^ In addition to SMP composites with nano‐fillers, researchers also studied actuators based on dry‐spun CNT yarns by introducing foreign guests. Baughman et al. designed and fabricated a series of CNT yarn‐based torsional or tensile artificial muscles by yarn twisting and guest infiltration, which showed excellent performance such as fast, large‐stroke actuation under various stimulation modes.^[^ ^16^ ^]^ However, it is the dry‐spun CNTs yarn that dominates the actuation behavior, and since its axial expansion/contraction is enabled by fiber twisting and untwisting, generation of a high actuation stress is usually accompanied by a relatively low strain (e.g. <3%), whereas at large actuation strains the produced stresses are very limited. Recently, Li et al. utilized the coiled CNT yarn structure and reported composite actuators by wrapping a shell of liquid crystal elastomer (LCE) with good coil separability and torsional stability, producing actuation stresses up to 17.7 MPa,^[^ ^17^ ^]^ which also represents a promising direction that deserves further study. Given those important progresses, it is highly desirable to develop multifunctional actuator structures by low‐cost materials and scalable fabrication process. Especially, for SMP‐based composite films or fibers, their actuation performance and deformation ability need to be optimized and integrated, and the mechanism including the critical role of nanofillers should be analyzed in‐depth.

Here, we studied fiber‐shaped actuators made from a conventional SMP polymer, polyurea, and a classical nanofiller, multiwalled CNTs, considering the low cost of materials and subsequent scalable fabrication of composites (for example, by wet spinning). Also, we can tailor the microstructure of polyurea/CNT (PU/CNT) composites, their mechanical and electrical properties, as well as their actuation performance. We adopted a heat‐assisted densification step to improve the structure and promote the role of CNTs in creating strong interaction with PU and improving the mechanical properties of resulting PU/CNT fibers. We demonstrated exceptional actuation performance both under thermal and electrical stimulation, as well as versatile shape memory behavior ranging from 1D to 3D shape changes. The high output performance and predesigned shape memory properties of PU/CNT fiber demonstrate wide potential fields such as soft robotics and aerospace engineering.

## 2 Results and Discussion

### 2.1 Preparation and Structure Evolution of PU/CNT Composite Fibers

**Figure 1:**
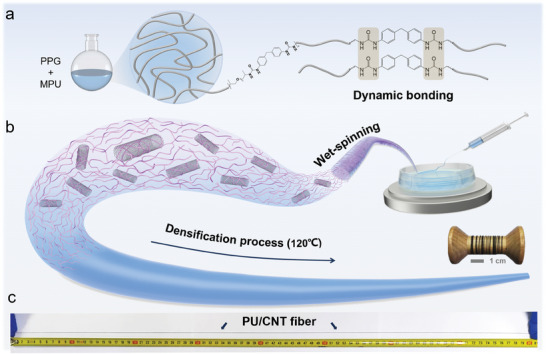
Preparation of PU/CNT composite fibers combining wet‐spinning with thermal densification. a) Schematic diagram of the synthesis of PU by combining a flexible backbone polymer (polypropylene glycol, PPG) with strongly oriented hydrogen bonding units (methylene biphenyl urea, MPU). b) Illustration of the fiber fabrication process using wet spinning followed by thermal annealing at a suitable temperature. The inset (right) is a digital photograph of PU/CNT fibers collected around a roller. c) Digital image of a meter‐long PU/CNT fiber with uniform diameter after annealing.

**Figure 2:**
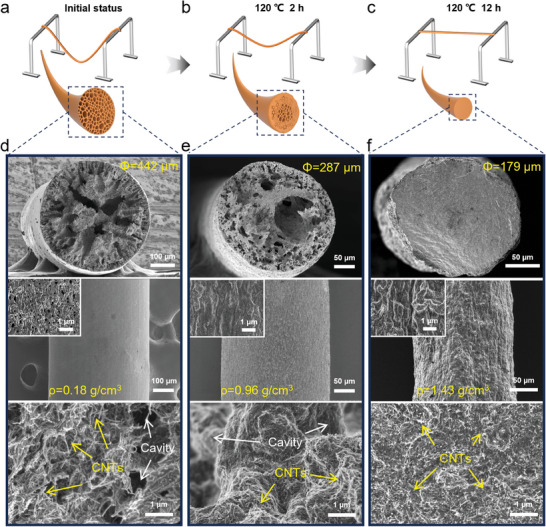
Structural characterization of PU/CNT fibers during thermal densification. a–c) Illustration showing a PU/CNT fiber suspended across a metal frame for thermal densification, and the axial shrinkage over time, as well as the illustrations of corresponding microstructure. d–f) SEM images showing the structural evolution in the PU/CNT fibers, including the cross‐section (top), surface (middle), and inner part (bottom). Insets in the middle row show high‐magnification SEM images of the fiber surface. Arrows in the bottom images point to the inner cavities and CNTs uniformly distributed through the PU matrix.

The PU/CNT fibers were prepared via wet spinning and heat‐assisted densification using shape memory PU (*M*_n_ = 43.7 kDa, *M*_w_ = 82.0 kDa, PDI = 1.88, Figures S1 and S2, Supporting Information) and powder‐form multiwalled CNTs. PU contains numerous hydrogen bonding units, and a tensile strain can induce the formation of supramolecular structures through oriented hydrogen bonding (**Figure** **1**a).^[^ ^18^ ^]^ CNTs are used as the nanofillers due to their 1D tubular structure and anisotropy, which increase the physical molecular cross‐linking density during tensile deformation and impact the mechanical and actuation properties of the PU/CNT fibers. The schematic diagram of the PU/CNT preparation process is shown in Figure 1b. A certain amount of CNTs were uniformly mixed with the PU solution and injected into a coagulation bath to obtain continuous PU/CNT fibers with tunable diameter (Figure S3, Supporting Information). And the CNTs in both the spinning solution and the composite fibers were uniformly and stably dispersed, which is confirmed by particle size analysis and SEM characterization (Figure S4, Supporting Information). Here, we adopted a thermal annealing step to densify the as‐spun fibers and obtain enhanced mechanical properties. To do this, a wet‐spun fiber was suspended over a frame (with a span of about 30 cm) with the two ends of fiber fixed at the contact points with the frame, and then subjected to high‐temperature treatment for a controlled period (120 °C, 0 to 12 h). We observed that the fibers shrank considerably (radial shrinkage of ≈46% and axial shrinkage of ≈36%) after thermal treatment, and the arc‐shaped fiber was straightened due to the significant longitudinal shrinkage (**Figure** **2**a–c). We think this suspending and straightening process is very helpful to fabricate strong fibers with uniform cross‐section, as the fixed ends would exert tensile force on the fiber during its shrinkage. If instead the fiber was placed on a substrate surface and allowed to shrink freely during thermal annealing, the shrinkage degree would be less and also it is easily to collapse into a ribbon‐like structure.

The corresponding microstructure evolution was revealed by scanning electron microscope (SEM) characterization. A wet‐spun fiber after natural drying (without heat‐assisted densification) shows a relatively large diameter of 442 µm, a smooth surface, and large cavities inside (Figure 2d). After thermal treatment for a short period (2 h), the fiber diameter decreases to 287 µm and there are still many pores with different sizes present internally (Figure 2e). After a sufficient treatment period (12 h), the fiber diameter further decreases to 179 µm and the residual pores completely disappeared, resulting in a fully densified cross‐section (Figure 2f). At the same time, the fiber surface becomes more rough due to the significant radial shrinkage, with a large‐degree increase in the fiber density (from initially ≈0.18 g cm^−3^ to currently ≈1.43 g cm^−3^). The presence of large amount of inner pores is due to the solvent exchange in the coagulation bath, and these pores must be removed completely in order to achieve superior mechanical and electrical properties. Thermal annealing at an appropriate temperature (120 °C, higher than the glass transition temperature (*T*_g_) of PU) enables the PU matrix to melt and fuse, whereas the internal pores were gradually removed during the period (Figures S5 and S6, Supporting Information). In addition, enlarged view of SEM images clearly shows the uniformly dispersed CNTs embedded in the PU matrix, forming close contact between their interfaces (Figure 2d–f, bottom). Such uniform distribution and interconnection of CNT fillers at relatively high loadings (here is 16.6 wt%) throughout the PU matrix ensure effective mechanical reinforcement as well as the formation of penetrated conductive paths for electrical actuation.

### 2.2 Mechanical Testing and Analysis on Critical Roles of CNTs

**Figure 3:**
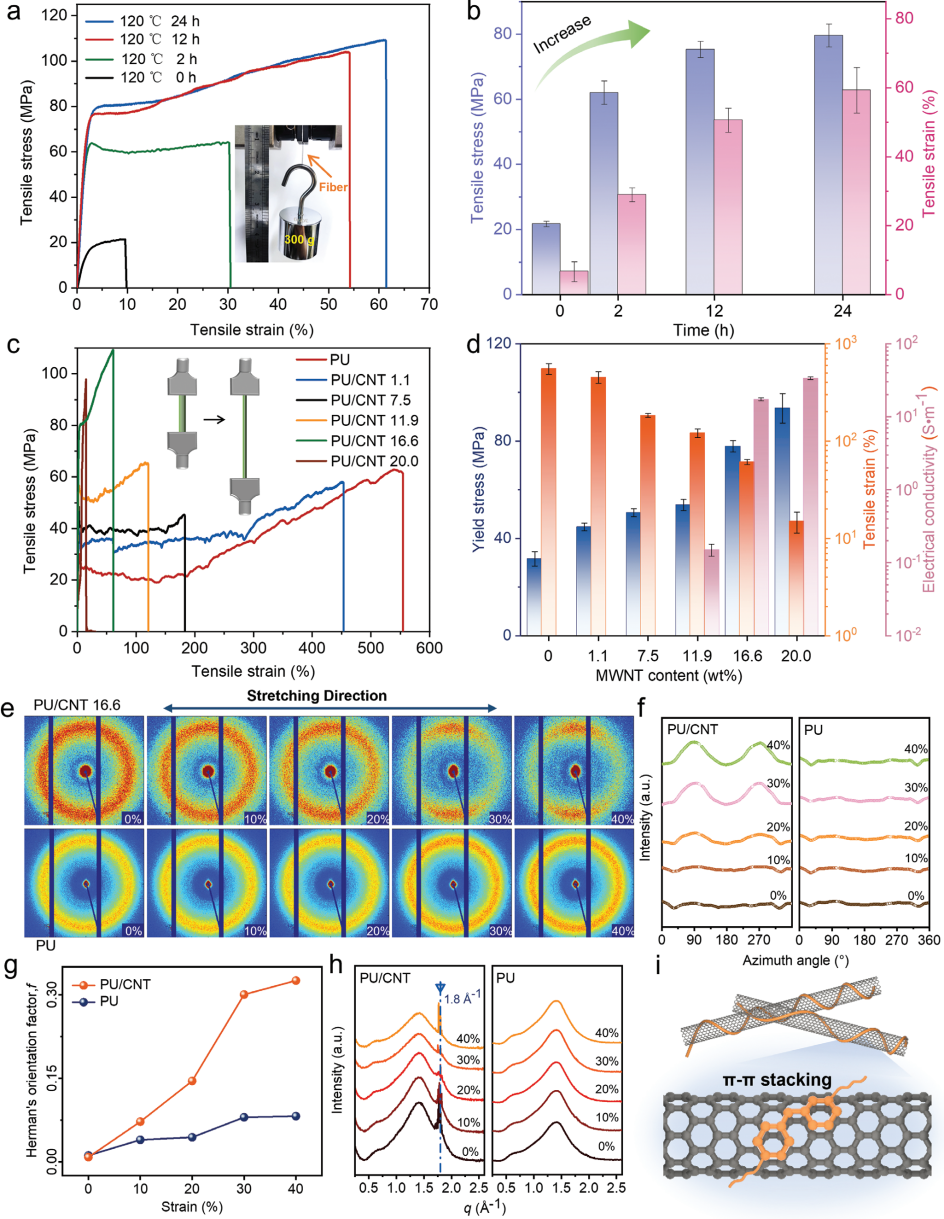
Mechanical properties of PU/CNT fibers and analysis of CNT roles. a) Tensile stress‐strain curves of PU/CNT fiber samples prepared by different thermal densification durations (0 to 24 h). Inset photo shows a PU/CNT fiber lifting a 300 g weight. b) Plot of yield stresses and fracture strains of fiber samples showing the increase and saturation of yield stress with longer annealing time (the average and error bar of each data point are plotted from 3 fiber samples). c) Stress‐strain curves of neat PU and PU/CNT fibers with different CNT loadings from 0 to 20.0 wt% (The notation of PU/CNT 1.1 represents the fiber sample with a CNT loading of 1.1 wt%). Inset shows a diagram of fiber stretching. d) Yield stress, strain, and conductivity of PU/CNT fibers with different CNT loadings (0–20 wt%). The electrical conductivity can be measured when the CNT loading was above 11.9 wt%. e) In situ 2D‐WAXS plots of a neat PU and a PU/CNT fiber (CNT loading is 16.6 wt%) in their original state and when they are stretched to different strains (0 to 40%). f) Corresponding 1D scattering mapping for azimuthal integration of the 2D WAXS data. g) Calculated Herman orientation factors (*f*) of the PU and PU/CNT fibers at different strains. h) 1D‐WAXS patterns of different assembly domains, where the signal at 1.8 Å^−1^ appeared in PU/CNT fibers corresponds to π–π interactions. i) Schematic of molecular π–π stacking between the aromatic rings in PU chains and the hexagonal rings at the CNT surface.

We have measured the tensile properties of our PU/CNTs fibers and systematically investigated the influence of key parameters such as the thermal annealing time (120 °C, from 0 to 24 h) and the CNT loading (0−20 wt%) on the tensile strength and fracture strain. Tensile stress–strain curves show that the pristine fiber before thermal treatment is relatively weak, and thermal annealing leads to significant increase in both the strength and strain due to the densification effect (**Figure** **3**a,b). The yield strength of the PU/CNT fiber (16 wt% CNT) after 12 h annealing, at which stage we observed complete removal of pores, reaches 78 MPa and the fracture strain is 59%. A single such fiber (3 cm in length) could lift a weight of 300 g, 3 × 10^5^ times heavier than its own weight, with a corresponding tensile stress of 62 MPa (inset of Figure 3a). Further annealing for longer time (24 h) does not lead to much change in the yield strength and strain. The thermal and electrical conductivities of the composite fibers were also improved after thermal densification (Figure S7, Supporting Information). After densification, the pores in the fibers shrank and disappeared, and the network of interconnected CNTs within the fibers increased, enabling efficient electron transport. As a result, the thermal conductivity of the fibers increased to 0.26 W m^−1^ K^−1^, 2.6 times higher than before densification (0.1 W m^−1^ K^−1^). Additionally, electrical conductivity was determined by measuring the dimensions and resistance of the composite fibers, which increased from 0.69 to 18.6 S m^−1^ after densification. On the other hand, as the CNT loading increases from 0 to 20 wt% (specific weight percentages were determined by thermal gravimetric analysis, Figure S8a, Supporting Information), the yield strength of the fiber increases whereas the fracture strain decreases monotonically (Figure 3c,d). It indicates that the loading of CNTs effectively improves the fiber strength, yet the tensile strain also decreases from 550% in pure PU fiber to only 15% in the fiber with 20 wt% CNTs. Also, we measured the electrical properties of the composite fibers, and found that the fiber became conductive when the CNT loading was more than 11.9 wt% (Figure S8b, Supporting Information). In this regard, here we selected the PU/CNT fiber (16.6 wt% CNT) since it has both high strength, good ductility and CNTs have formed conductive paths within the fiber.

Furthermore, the presence of CNTs plays multiple important functions in tailoring the microstructure of composite fibers. On one hand, the addition of CNTs promotes the orientation of polymer chains upon stretching. On the other hand, the hexagonal ring structure of CNTs forms π–π interactions with the phenyl rings of the polymer, further enhancing the fiber strength. On the molecular scale, PU can fix their shape during stretching by hydrogen bonding orientation (in a direction perpendicular to the fiber axis) at room temperature. Similar to PU fibers, CNT/PU exhibits such properties and the incorporation of CNTs contributes to the tension‐induced orientation of the polymer chains. The orientation change of PU/CNT fibers (16.6 wt%) during stretching was characterized by in situ 2D wide‐angle X‐ray scattering (WAXS) (Figure 3e). This 2D‐WAXS method has been used to study the polymer chain orientation previously at the molecular level.^[^ ^19^ ^]^ During in situ tensile testing, as the tensile strain of PU/CNT fibers gradually increased from 0% to 40%, a distinct split in the 2D‐WAXS pattern appeared at 30% strain, compared to pure PU fibers where it emerged only at 50% strain (Figure S9, Supporting Information). Under a relatively high loading of CNTs, the PU matrix can be seen as being uniformly mixed/inserted in the confined space between CNTs, which leads to much enhanced PU chain orientation even at a moderate strain. The increased orientation level in the composite fibers was mainly attributed to the cold‐drawing process, underscoring the role of embedded CNTs in promoting polymer chain alignment. Figure 3f, which represents the 1D scattering of the azimuthal integral of 2D‐WAXS data, further confirms this result. The fiber orientation was quantified by the Hermans orientation factor, which increases with tensile strain consistently (Figure 3g). Compared with pure PU fibers, the orientation level of the PU/CNT is much enhanced. The 1D X‐ray diffraction pattern in Figure 3h reveals the interactions at the molecular scale within the fibers.^[^ ^20^ ^]^ The signal in the range of 0.6 Å^−1^ corresponds to hydrogen bonds in the polymer, while the PU/CNT exhibits an additional strong signal at 1.8 Å^−1^, corresponding to a distance of 0.34 nm. While the multiwalled CNTs with such a layer distance could contribute to this signal, we also believe that there should be extensive π–π interactions between CNTs and the polymer phenyl rings in our well‐mixed composite fiber. We also confirmed this through Raman characterization of the fibers (Figure S10, Supporting Information). The interaction between CNTs and the polymer is reflected in the Raman band shift or changes in bandwidth.^[^ ^21^ ^]^ Compared to pure CNTs powder, the two typical bands of composite fibers showed slight band shift. In comparison to the untreated composite fibers, the D and G bands of CNTs in the densified composite fibers upshifted (by 2 and 3 cm^−1^, respectively), indicating that CNTs were under compressive forces from the surrounding PU. It also indicates strong interfacial interactions between the PU matrix and CNTs. Due to the presence of benzene rings in the PU structure, it could be considered that this interfacial interaction was the result of PU self‐assembling on the CNTs surface through π–π stacking. A schematic representation of the π–π interactions formed by the entanglement between polymer chains with CNTs is illustrated in Figure 3i.

### 2.3 Thermal Actuation Properties of PU/CNT Fibers

**Figure 4:**
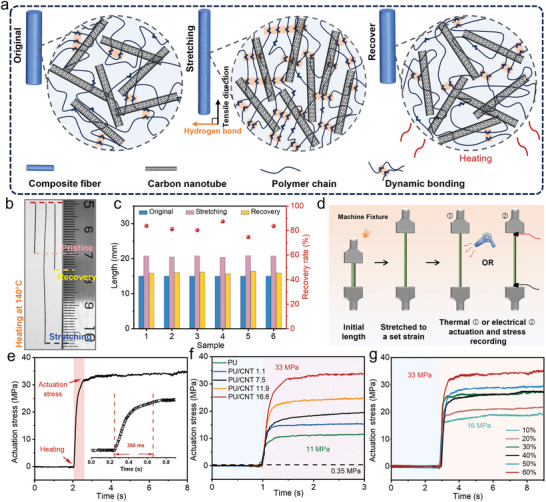
Thermal actuation properties of PU/CNT fibers. a) Microscopic illustration of the strain recovery process of PU/CNT fibers under thermal stimulation. b) Photo of a PU/CNT fiber in its initial state (20 mm in length), stretched and temporarily fixed state at 170% strain (54 mm), and recovered state upon heating (26 mm). c) Length change of multiple fibers before and after strain recovery experiments and their corresponding recovery rates. d) Schematic of in situ testing of fiber actuation stress by a lab set‐up. e) Recorded thermal actuation stress in a prestretched (to a strain of 60%) PU/CNT16.6 fiber, and inset shows a magnified view of the stress increase portion. f) Thermal actuation stress of PU and PU/CNT fibers (with different CNT loadings) at 60% pre‐strain. The horizontal dashed line represents the actuation stress (0.35 MPa) of human skeletal muscles. g) Thermal actuation stresses in PU/CNT16.6 fibers tested at different pre‐strain levels (10%–60%).

The stress output of PU/CNT when subjected to external thermal stimuli is one of the most important measures of its actuation performance.^[^ ^22^ ^]^ The microstructural schematic of the PU/CNT for pre‐defined tensile deformation and thermally stimulated recovery is shown in **Figure** **4**a. The anisotropic CNTs in pristine PU/CNT fiber are randomly arranged. When the fiber is stretched to a certain strain, the CNTs and polymer chains move with the strain, and some of the hydrogen bonds between the polymer chain segments are formed and fixed, leading to temporarily fixed stretched state (Figure S11a, Supporting Information). When thermal stimulation is applied to dissociate the hydrogen bonds between the molecular chains, the strain of the fiber is released, and the internal CNTs and polymer chains return to their original disordered state.^[^ ^23^ ^]^ The corresponding macroscopic shape memory process of one of the fiber samples (PU/CNT1.1) is depicted in Figure 4b. The fiber has an original length of 20 mm, and was stretched to a temporary strain (170%) and fixed at that length (54 mm) at room temperature. Upon external heating, the fiber quickly shrank back to a length of 26 mm, corresponding to an actuation strain of 82%. To examine the uniformity of the wet‐spun fibers, we recorded the lengths of multiple samples in initial state (all cut into 15 mm length), stretched temporarily fixed state, and recovered state after thermal actuation (Figure 4c). The recovery rates of the fibers (defined as the ratio of recovered length during actuation over stretched length) are generally maintained at around 80%. The recovery rate of shape memory polymers with good shape memory effects was mostly around 90% or higher.^[^ ^24^ ^]^ Although the addition of CNTs to PU reduced the shape recovery rate of PU, it significantly improved the mechanical and actuation performance of the composite.

Next, we tried to directly measure the output stress during the actuation of PU/CNT fibers by an in situ mechanical testing setup (as illustrated in Figure 4d). The fiber sample was clamped straightly by two grips in the setup and then stretched by the top grip to a set strain where the fiber was fixed due to the effect of hydrogen bonding at room temperature. Then thermal or electrical actuation was triggered and the stress produced by the fiber (which was maintained at the stretched state but tends to shrink) was recorded over time. For one of our optimized fiber sample (PU/CNT16.6) which was prestretched to a strain of 60% (Figure S11b, Supporting Information), thermal actuation produced a maximum stress value of 33 MPa stably, and the stress increased very rapidly (within 360 ms) (Figure 4e). Using this test method, the actuation stress curves of PU/CNT with different CNT loading at the same pre‐strain of 60% are summarized in Figure 4f. Compared to the pure PU fiber, the actuation stress of PU/CNT increased consistently with increasing CNT loading, and was much superior to the performance of most nanofiller‐reinforced SMP composites (typically within the range of 0–10 MPa). For the composite fiber of PU/CNT16.6, its actuation stresses under different pre‐strains were compared, revealing that the greater the prestrain, the greater the actuation stress (Figure 4g). This may be due to that the polymer chains are more stretched at higher strains, leading to the formation of an increased number of intermolecular hydrogen bonds, which enhances the actuation stress when these bonds are released upon heat stimulation.

### 2.4 Electrical Actuation Properties of PU/CNT Fibers

**Figure 5:**
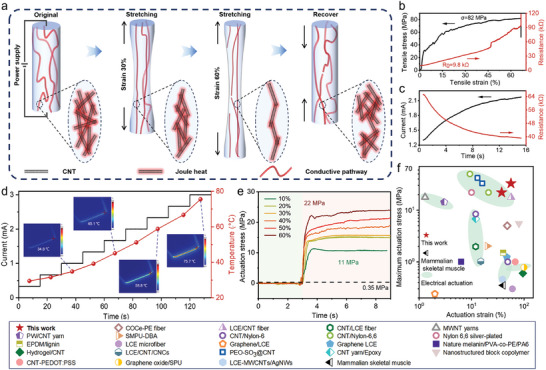
Electrical actuation properties of PU/CNT fibers. a) Schematic of change in CNT conductive pathways during stretching and recovery processes of PU/CNT fibers. b) Changes in stress and electrical resistance of the PU/CNT16.6 fiber during tensile testing. c) Changes in current and resistance of a shrink prestrained (60%) PU/CNT fiber under constant voltage. d) PU/CNT16.6 produces different temperature changes under different current stimulation. Insets show infrared images of the fiber passing electrical current of 1, 1.7, 2.3, and 3 mA, respectively. e) Electrical actuation stresses corresponding to a pre‐stretched PU/CNT16.6 at different strains. f) Comparison of actuation stresses (thermal/electrical) between PU/CNT fibers and recently reported composite materials.

Our PU/CNT composite fibers consist of uniformly distributed CNTs which could form a percolated network throughout the PU matrix, rendering the composite to be electrically conductive at a sufficient CNT loading (Figure S11c, Supporting Information). Moreover, the embedded CNTs could deform with the PU matrix; when the fiber was stretched, the CNTs maintain the conductive network until a certain tensile strain (60%) although the interconnected paths would decrease upon stretching. When the fiber recovers to nearly its original length, the CNT network also recovers its interconnection. Such a mechanism makes it possible for electrical actuation of PU/CNT fibers, by utilizing the Joule heat transferred from the percolated CNT network to the surrounding PU matrix (as seen in **Figure** **5**a). To demonstrate this, we studied the electrical behavior of the composite fiber coupling its mechanical deformation. First, the electrical resistance was monitored during the tension process, which showed a monotonic increase with the strain until fiber breakage, in consistent with our analysis (Figure 5b). Second, a PU/CNT fiber was prestretched a 60% strain, and a constant voltage was applied through the fiber ends which stimulated the shape recovery process. We observed that the current gradually increased, indicating that the resistance dropped when the fiber was shrinking (recovery of interconnected CNT paths) (Figure 5c).

The electrically induced heating effect was revealed by monitoring the temperature change along the fiber using an infrared camera. When the current value was increased to 3 mA, the temperature on fiber body reached 75.7 °C, exceeding the *T*_g_ temperature of the composite fibers (Figure 5d and Figure S11d, Supporting Information). *T*_g_ of the fiber was determined using differential scanning calorimetry (DSC). The results showed that the *T*_g_ of the PU/CNT fibers was lower than neat PU fibers. We believe that the decrease in *T*_g_ is primarily due to the incorporation of CNTs, which disrupt the stable hydrogen bond structure of the polymer. And the presence of a small amount of residual solvent acting as a plasticizer may also contribute to this effect. Using the same setup in Figure 5e, we measured the output stress in the PU/CNT16.6 fiber pre‐stretched to different strains (10% to 60%) during electrical actuation. The actuation stress of the fiber was enhanced substantially with increasing strain, reaching a value as high as 22 MPa at a pre‐strain of 60%, and was released within a short period (less than 1 s).

Figure 5f collects maximum actuation stress and actuation strain data for human skeletal muscle and composite actuators reported in the literature (Table S1, Supporting Information).^[^ ^6^, ^7^, ^11^, ^12^, ^16^, ^17^, ^25^ ^]^ Thermal response SMP typically achieved electrical actuation by incorporating conductive fillers such as graphene, carbon black, CNTs, etc. However, the actuation stresses of directly molded composite actuators (including bulk, thin film, and fibers) are generally low (typically not exceeding 10 MPa). Composite actuators made from CNT yarn exhibit higher actuation stresses, but the axial expansion/contraction of the composite material depends significantly on the twisting and untwisting of the fibers.

After that, to evaluate the cyclic stability of PU/CNT composite fibers, we conducted cyclic tensile tests, strain actuation cycles, shape actuation cycles, and recorded changes in actuation stress of the fibers. First, we performed cyclic tensile tests at a strain of 10% (Figure S12a, Supporting Information). During the repeated tensile cycles, the fibers exhibited structural relaxation, and the residual strain gradually increased. This was attributed to the slip of polymer chains generated in the composite fibers during the tensile process. After 200 cycles, the stress decreased by approximately 30%. The changes in the aspect ratio of the fibers during the cyclic thermal recovery were also measured (Figure S12b, Supporting Information). All samples (pure PU, PU/CNT7.5, and PU/CNT16.6) exhibited a certain degree of plastic deformation, with the fibers tending to become longer and thinner, and the aspect ratio gradually increasing. The addition of CNTs negatively impacts the repetitive actuation cycles. During thermal recovery, CNTs hinder the polymer chains from returning to their initial state, with higher CNT content making it more difficult for the chains to recover. Additionally, the shape actuation cyclic performance of the composite fibers was evaluated. The composite fibers, preprogrammed into a hollow spring shape, maintained their temporary shape at room temperature and then recovered under thermal stimulation. Figure S12c (Supporting Information) shows the changes in length and recovery rate of the spring‐shaped fibers under 120 °C thermal stimulation. The PU/CNT fibers sustained 20 cycles of shape recovery. With the increase of cycles, the recovery rate gradually decreased until breakage during the 21st stretching cycle. The cause of breakage was likely due to the inevitable formation of small cracks in the composite fibers during each stretching, which could not be repaired during thermal recovery, resulting in an accumulation of cracks and eventual fiber breakage. The repeatability of actuation was further quantitatively evaluated using actuation stress (Figure S12d,e, Supporting Information). With prestrain levels ranging from 10% to 60%, the initial actuation stress was greatly enhanced, but the composite fibers with high prestrain experienced severe stress degradation and poor repeatability during the thermal recovery cycling. Each thermal recovery induced a certain degree of plastic deformation. This affected the number of hydrogen bonds formed in subsequent stretching cycles, thereby resulting in the decline of output stress. Furthermore, the greater the prestrain, the more likely the composite fibers were to develop crack defects, resulting in fewer recovery cycles.

### 2.5 Applications of PU/CNT Fibers in Weight Lifting and Programmed Shape Deformation

**Figure 6:**
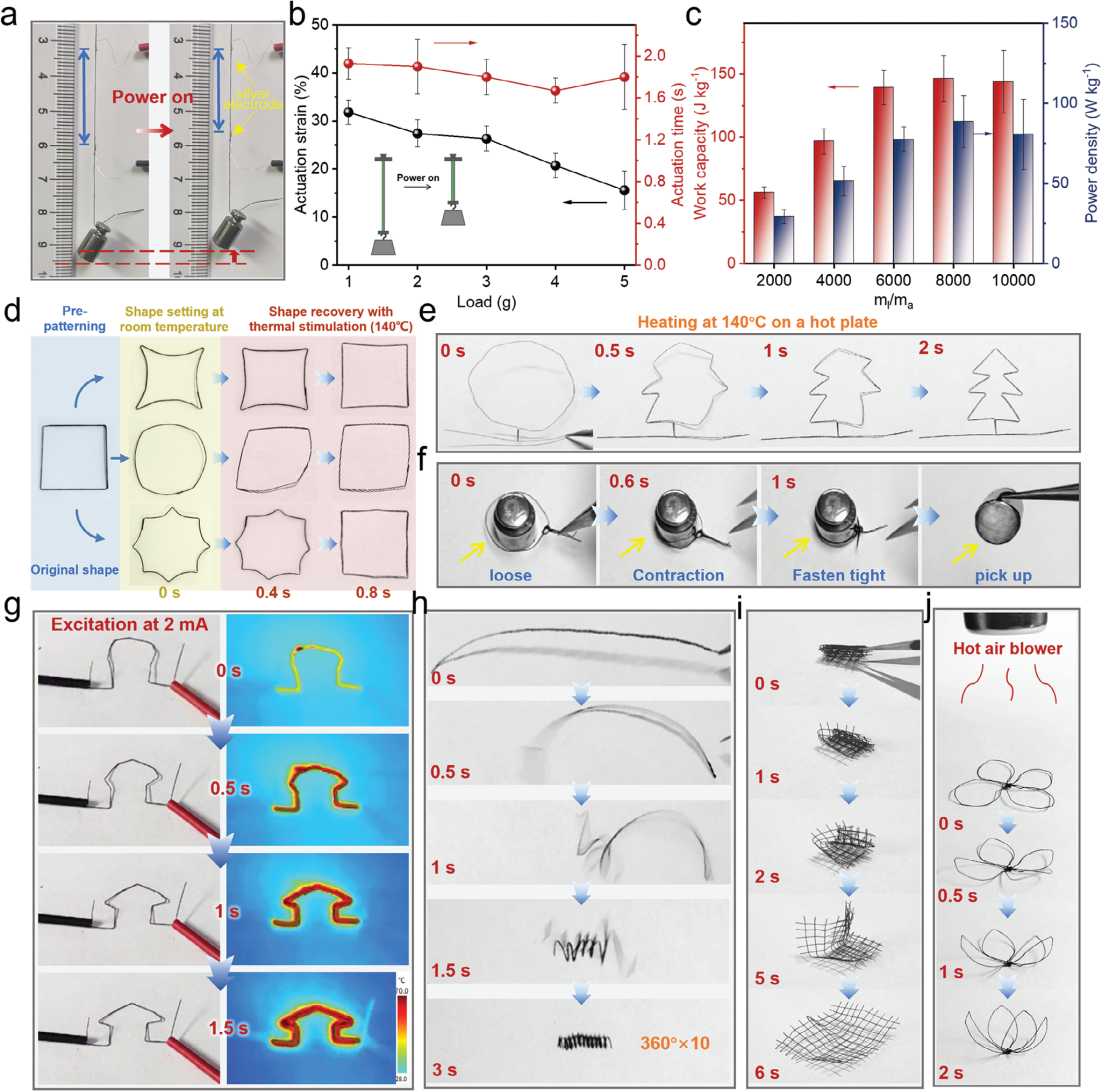
Demonstration of PU/CNT Fibers in weight lifting and programmed shape deformation. a) Photos of a PU/CNT fiber lifting a weight of 2 g by electrical actuation. Power is applied through the silver wires at the fiber ends. b) Measured actuation strains and time for lifting different loads (1–5 g) by PU/CNT fibers. Inset shows a schematic of fiber lifting. c) Calculated work capacities and power densities of PU/CNT fibers. d) Photographs of simple shape change (curvature, angle) in PU/CNT fibers (pre‐designed into a rectangle). e) Sequential snapshots of shape change in a PU/CNT fiber back to a pine tree shape on a 140 °C hot plate. f) A series of photos showing a pre‐strained fiber loop fastening tightly around and lifting a 2 g weight under thermal stimulation. g) Sequential snapshots of shape change into a house‐like structure under electrical stimulation, and corresponding infrared thermal images. h– j) Sequential snapshots of 3D shape transformation in pre‐designed PU/CNT fibers (or fabric) such as a hollow spring, a folded mesh and a flower.

Finally, we demonstrate potential applications of our PU/CNT fiber actuators in weight lifting and programmed shape deformation, in both thermal and electrical actuation. For example, we adopted electrical excitation on prestrained (60%) PU/CNT16.6 fibers (by passing a current of 2 mA through the fiber), and found that the fiber could contract and lift weights ranging from 1 to 5 g (equivalent to 2000–10 000 times their own weight) (**Figure** **6**a). The fibers generally can lift the 1 g weight upward by a maximum actuation strain of 32%, and even the weight is increased to 5 g, the fibers could still produce actuation strains of about 16%. Also, all tested fiber samples were able to quickly lift the weights within 2.2 s as observed through video analysis (Figure 6b). Based on the weight lifting experiments, we could calculate the work capacity and power density of our PU/CNT fibers under electrical actuation, which were also important metrics for evaluating actuator performance. The work capacity and power density varied with changing load mass, and the maximum values (at a load mass of 8000) reached 146 J kg^−1^, and 89 W kg^−1^ respectively, both surpassing natural mammalian muscles (39 J kg^−1^, 50 W kg^−1^) (Figure 6c). In addition to electrical actuation, thermal actuation resulted in a maximum strain of 56%, and also high work capacity (206 J kg^−1^) and energy density (106 W kg^−1^) (Figure S13, Supporting Information).

The shape memory process accompanied by the axial contraction in the PU/CNT fibers allows us to design versatile shape transformations in 2D and 3D configurations, utilizing the robustness, flexibility, and weavability of our composite fibers. Unlike traditional SMP, the predesigned patterns could be directly fixed at temporary shapes by room temperature manipulation, owing to the formation of dynamic bonding connections upon stretching within PU/CNT fiber. Fibers were designed as self‐supporting structures in multiple dimensions, allowing for controlled spatial deformation under external thermal or electrical stimuli. First, based on a closed rectangle created by a single fiber, we demonstrated 2D shape changes between different curvatures and angles (Figure 6d). The rectangles were transformed and fixed at room temperature into various temporary shapes by pushing the 4 edges inward (negative curvature), or pulling the edges outward into a circle (positive curvature), or pulling the middle points of edges outward to form an octagons (thus creating an acute angle in each edge). Significant shape transformations were observed under thermal stimulation (140 °C), with the overall change of three patterns (ultimately returning to the rectangular shape) completed within 0.8 seconds. Curvature and angle changes were attributed to subtle deformations at specific points (e.g., corner or middle of the edge) in the PU/CNT fiber rectangle, resulting in different shape changes at the macroscopic level. Also, a relatively complex pattern resembling a “pine tree” was realized from a temporary circular shape within 2 s upon heating (Figure 6e). Further, a PU/CNT fiber was stretched and made into a loop surrounding a metal cylinder (2 g); upon heating, the initially loose fiber loop shrank and tightly fastened the cylinder, which could lifted afterward (Figure 6f). The above shape transform also could be realized by electrical stimulation, for example, we directly pass a small current (2 mA) through the pre‐patterned PU/CNT fiber, and changing it back to the house‐like shape due to efficient Joule heating effect (within 1.5 s) (Figure 6g). Uniform heating during the shape memory process was observed through fiber surface temperature measurements using an infrared camera.

Moreover, we demonstrate more controlled shape change behavior by designing 3D fiber patterns for spatial transformation. For example, a PU/CNT fiber was preprogrammed into a hollow spring, which was straightened and fixed at room temperature (Figure 6h). When placed on a hot plate (140 °C), the fiber underwent continuous twisting in 3D space to recover its spring shape, achieving an overall rotation of approximately 3600° within a deformation time of 3 s. These fibers also could be woven into a mesh fabric and folded, and upon heating, the fabric could open in a reversed folding sequence and recover the initial flattened shape (Figure 6i). Such fabric opening process represents the shape transformation from 3D (stacked structure) to 2D, and the shape change from 2D to 3D also could be realized. For example, several fibers were woven into a flat flower‐like pattern with 4 elliptical petals, and when triggered by heat, the petals in a temporarily fixed pattern raised upward to form a 3D flower within 2 seconds (Figure 6j). These examples demonstrate the application potential of our PU/CNT fibers in making innovated actuators with versatile shape transformations (Figure S14 and Movies S1–S3, Supporting Information).

## 3 Conclusion

In this paper, nanocomposite fibers were fabricated by wet‐spinning with heat‐assisted densification method. The role of CNTs within densely packed composite fibers was pivotal. Firstly, CNTs promoted the stretching orientation of the fibers. And then the strong interaction between the nanofiller CNTs and PU resulted in a good interfacial combination. Both of these factors contributed to the enhancement of fiber mechanical, electrical, and output performance. In thermal actuation, PU/CNT fibers showed actuation stresses ranging from 11 to 33 MPa and demonstrated a rapid response. Electrical actuation achieved an actuation stress of 22 MPa. The output performance exceeds that of most previously reported SMP‐based composite actuators (polymers with fillers such as CNTs, graphene, carbon black, etc.). Furthermore, the PU/CNT fibers demonstrated preprogrammed spatial deformation properties through various shape changes, including transformations between 1D, 2D, and 3D shapes. Therefore, this fiber actuator shows significant potential for applications in soft robotics, wearable electronic devices, aerospace engineering, and other fields.

## 4 Experimental Section

### Materials

CNT powder (purchased from Jiangsu Cnano Technology Co., Ltd.), nitric acid, N,N‐dimethylformamide, MPU, PPG (molecular weight: 400), dichloromethane, n‐hexane, methanol, deionized water. All chemicals were used as received.

### Preparation of Polyurea

MPU and PPG were separately dissolved in dichloromethane. The two solutions were then mixed and stirred for 72 h at 50 °C under an argon atmosphere until the solution gelled and partially precipitated. A small amount of methanol was added to quench the reaction, followed by the addition of excess hexane to precipitate the material thoroughly. The collected material was then dried completely in a vacuum oven.

### Preparation of PU/CNT Composite Fibers

CNTs were immersed in nitric acid for acidification at 120 °C for 12 h. Afterward, the solution was taken out, washed with deionized water, and freeze dried. A small amount of CNT powder was dispersed in the organic solvent N, N‐dimethylformamide (5 mL) using ultrasonication. After uniform dispersion, polyurea (0.6 g) was added and stirred to dissolve. Subsequently, the remaining CNT powder was added. Taking the example of a 20 wt% composite fiber, the total amount of CNT added in two steps was 0.15 g. The spinning solution was stirred and transferred to a syringe and then injected into a coagulation bath (deionized water) through a 21 G needle (inner diameter 0.51 mm) at an injection pump speed of 10 mL h^−1^. After soaking the fiber in the bath for five minutes, it was removed and transferred to a suspended drying process in an oven at 120 °C.

Considering the scalability of the wet‐spinning process and its economic viability for large‐scale production, we further estimated raw materials cost. However, some potential issues need to be addressed for large‐scale production (Note S1, Supporting Information).

### Measurement of Actuation Stresses

The actuation stress was tested on a single‐column tester. The tensile speed was set to 15 mm min^−1^, and the sample diameter was accurately measured using a screw micrometer, with a fixed length of 15 mm. Regarding the actuation stress, the sample was first stretched to 0–60% of the prestrain, with the actual tensile strain slightly higher than the desired value (≈5%), to ensure complete fixation of shape at the target strain. After removing external forces, a heat gun or electrical current was used to provide stimulation, and the stress variations during the fiber induction process were fully recorded by single‐column tester.

### Measurement of Strain Actuation Cycles

Thermal recovery was cycled under 10% strain for pure PU fibers and composite fibers with 7.5% and 16.6% CNT content. The changes in length and diameter of the fiber were measured after each cycle.

### Measurement of Thermal Conductivity

PU/CNT16.6 sheet samples were prepared using the same method. Then, the thermal conductivity was measured using the transient hot wire method.

### Predesign of Pattern Deformation

Pattern deformation was classified into 1D, 2D, and 3D deformations. For 1D deformation, single‐column tester was used to stretch the material to the desired strain. For 2D and 3D deformations, such as rectangles, pine trees, and houses, simple 2D molds were used for shaping. Hollow spirals were formed by uniformly winding wire to create the hollow spiral structure. folded mesh involved overlapping different fibers together. Flower shapes were molded using specific molds. The processes demonstrated in the text all involved the recovery process after deformation caused by external forces. Additionally, the design of shapes was formed during the drying process, and temporary shapes (deformation caused by external forces) could be fixed at room temperature. The recovery of various shapes was completed on a heating plate at 140 °C, and spatially uniform heating provided by a heat gun (set at 140 °C) facilitated faster shape unfolding.

### Characterization and Measurement

Nuclear magnetic resonance (NMR) hydrogen spectra were used to determine the peak spectra of synthesized polyurea. Gel permeation chromatography (GPC) was used to determine the molecular weight of polyurea. Particle size analyzer (Zetasizer Nano ZSP) was used to determine the dispersion of CNTs in the predispersion solution. Thermal conductivity meter (TC3100D) was used to determine the thermal conductivity of the samples. Micro Raman imaging spectrometer (DXRxi) was used to determine the interactions between CNTs and polymers. Scanning electron microscopy (SEM) was used to observe the surface morphology and cross‐section of fibers. Thermal stability and content of host‐guest materials were characterized using a thermogravimetric analyzer under an air atmosphere. Additionally, DSC was conducted under a nitrogen atmosphere with a heating rate of 10 °C min^−1^ to measure the glass transition temperature (*T*_g_) of neat PU fibers and PU/CNT fibers.

The orientation of composite fibers and intermolecular interactions were determined using a small‐angle instrument with Cu λ as 1.542 Å X‐ray source, and wide‐angle X‐ray scattering was utilized to measure the orientation of PU/CNT composite fibers. Subsequently, orientation parameters *f* was calculated based on the Hermans‐Stein orientation distribution function.

(1) $$f\;=\frac{1}{2}\;\left(3\langle \mathrm{co}s^{2}\theta \rangle -1\right)$$

(2) $$\langle \mathrm{co}s^{2}\theta \rangle =\frac{\int_{0}^{\pi /2}I\left(\theta \right)\;\mathrm{co}s^{2}\theta \;\sin\theta \;d\theta }{\int_{0}^{\pi /2}I\left(\theta \right)\;\sin\theta \;d\theta }$$

where *I*(θ) is the intensity and θ is the azimuthal angle.

The mechanical properties of fibers were evaluated using a universal material testing machine (Instron 5843), including actuation stress for PU/CNT fibers with different loadings and strains. Electrical properties of fibers were tested using a Keithley 2400, which also provided power during electroactive actuation. Heat sources for shape recovery of predesigned patterns were provided by a hot plate or a hot gun. Temperature changes during the electrothermal process of composite fibers were determined using an infrared camera (Guide PS600). Dynamic mechanical analysis (DMA) was conducted under air atmosphere using DMA Q800 and DMA 850.

### Statistical Analysis

The samples tested for thermal/electrical actuation utilized PU/CNT16.6 fibers with an effective length of 20 mm and a diameter of approximately 180 µm (unless otherwise specified). The portions outside the effective length were used for securing the fibers and suspending weights. Each data point in the figure represents the average and error bars of three experimental measurements.

## Conflict of Interest

The authors declare no conflict of interest.

## Supporting information

Supporting Information

Supplemental Movie 1

Supplemental Movie 2

Supplemental Movie 3

## Data Availability

The data that support the findings of this study are available from the corresponding author upon reasonable request.
