# Supplementary material for: Wet‐Spinning Carbon Nanotube/Shape Memory Polymer Composite Fibers with High Actuation Stress and Predesigned Shape Change
Source: Adv Sci (Weinh). 2024 Aug 9;11(38):2404913. doi: 10.1002/advs.202404913 (PMC11481471; doi:10.1002/advs.202404913)
Supplement: Supplementary file 1 — Supporting Information [file ADVS-11-2404913-s003.docx]

**Supporting Information**

**Wet-Spinning Carbon Nanotube/Shape Memory Polymer Composite Fibers with High Actuation Stress and Pre-designed Shape Change**

Meng Li^1,2^, Kun Chen^2^, Ding Zhang^1^, Ziming Ye^2^, Zifan Yang^3^, Qi Wang^2^, Zhifan Jiang^2^, Yingjiu Zhang^1^, Yuanyuan Shang^1,^*, Anyuan Cao^2^*

^1^Key Laboratory of Material Physics, Ministry of Education, School of Physics and Microelectronics, Zhengzhou University, Zhengzhou 450052, China

^2^School of Materials Science and Engineering, Peking University, Beijing 100871, China

^3^Beijing National Laboratory for Molecular Sciences, Key Laboratory of Polymer Chemistry and Physics of Ministry of Education. Center for Soft Matter Science and Engineering, College of Chemistry and Molecular Engineering, Peking University, Beijing 100871, P. R. China

* Corresponding authors: yuanyuanshang@zzu.edu.cn and [anyuan@pku.edu.cn](mailto:anyuan@pku.edu.cn)

**Contents**

**Note S1.** Estimated raw materials cost.

**Note S2.** The safety of composite fibers.

**Figure S1.** ^1^H NMR labeled spectra of PU.

**Figure S2.** Gel permeation chromatography (GPC) trace of PU.

**Figure S3.** Additional characterization of PU/CNT16.6 fibers.

**Figure S4.** Dispersion of CNTs in spinning liquids and PU/CNT fiber.

**Figure S5.** SEM images of the PU/CNT fiber during the drying process.

**Figure S6.** Additional SEM images.

**Figure S7.** Changes in thermal and electrical conductivity of the PU/CNT fiber before and after densification.

**Figure S8.** Characterization of composite fibers with different CNT loadings.

**Figure S9.** 2D-WAXS plots of a neat PU fiber stretched to 50% strains.

**Figure S10.** Raman spectra of CNTs and composite fibers before and after densification.

**Figure S11.** Characterization of additional properties of the PU/CNT 16.6 fiber.

**Figure S12.** Cyclic actuation properties of PU/CNT fibers.

**Figure S13.** Demonstration of weight lifting by PU/CNT Fibers under thermal actuation.

**Figure S14.** Additional demonstration of pre-patterned PU/CNT fibers and their shape recovery process.

**Table S1.** Comparison of the actuation stresses among different composite actuators reported in literature.

**Supplementary Movies.**

**Note S1. Estimated raw materials cost.**

The precursor materials for the composite fibers are PU and CNT powder. PU is synthesized from polypropylene glycol (PPG) and methylene bisphenyl urea (MPU). The cost of PPG oligomer (0.4 kDa, amino-terminated, Jeffamine D400) is approximately $1,000 per 200 kilograms ($5 per kilogram). The cost of pure MDI is about $1,500 to $3,000 per ton (907 kilograms), which is less than $3.5 per kilogram. Noting that polymerization requires a 1:1 molar ratio of the two components, leads to an estimated raw materials (PU) cost of $4.35/kg. The price of CNT powder is approximately $7-14 per kilogram (less than $15 per kilogram). Based on a feed ratio of 4:1, the cost of the composite fibers is approximately $5.8 per kilogram. In addition to the raw material costs, large-scale production will also need to consider labor, equipment, energy, and other costs. ^[1]^

**Note S2. The safety of composite fibers.**

The PU/CNT fibers were not intended for biological applications, so the primary concerns were skin contact during use and potential CNT shedding. When using CNTs, protective measures such as wearing gloves, protective clothing, goggles, and working in a fume hood were taken to reduce exposure risks for operators. ^[2]^ Afterwards, CNTs were uniformly and stably embedded within the polymer matrix of the fibers. Stretching, bending, and breaking would not cause the CNTs to shed or become exposed.

At the end of the life of PU/CNT fiber, it could be safely reused and does not need to be sent to a landfill like other CNT materials. Since this composite fiber contains only PU and CNTs, it could be dissolved back into a spinning solution using the solvent DMF, thereby mitigating its environmental impact.


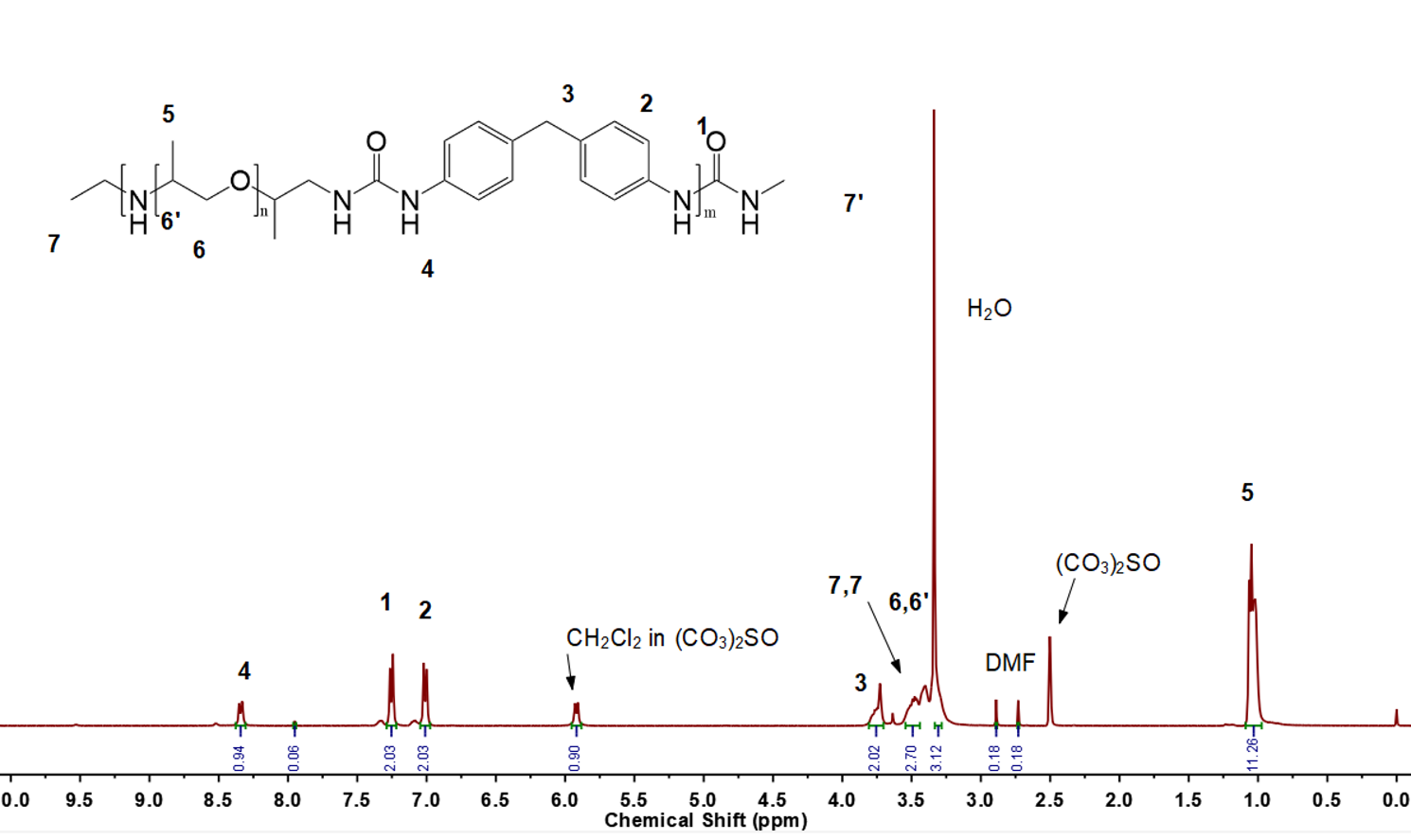


**Figure S1. ^1^H NMR labeled spectra of PU.**


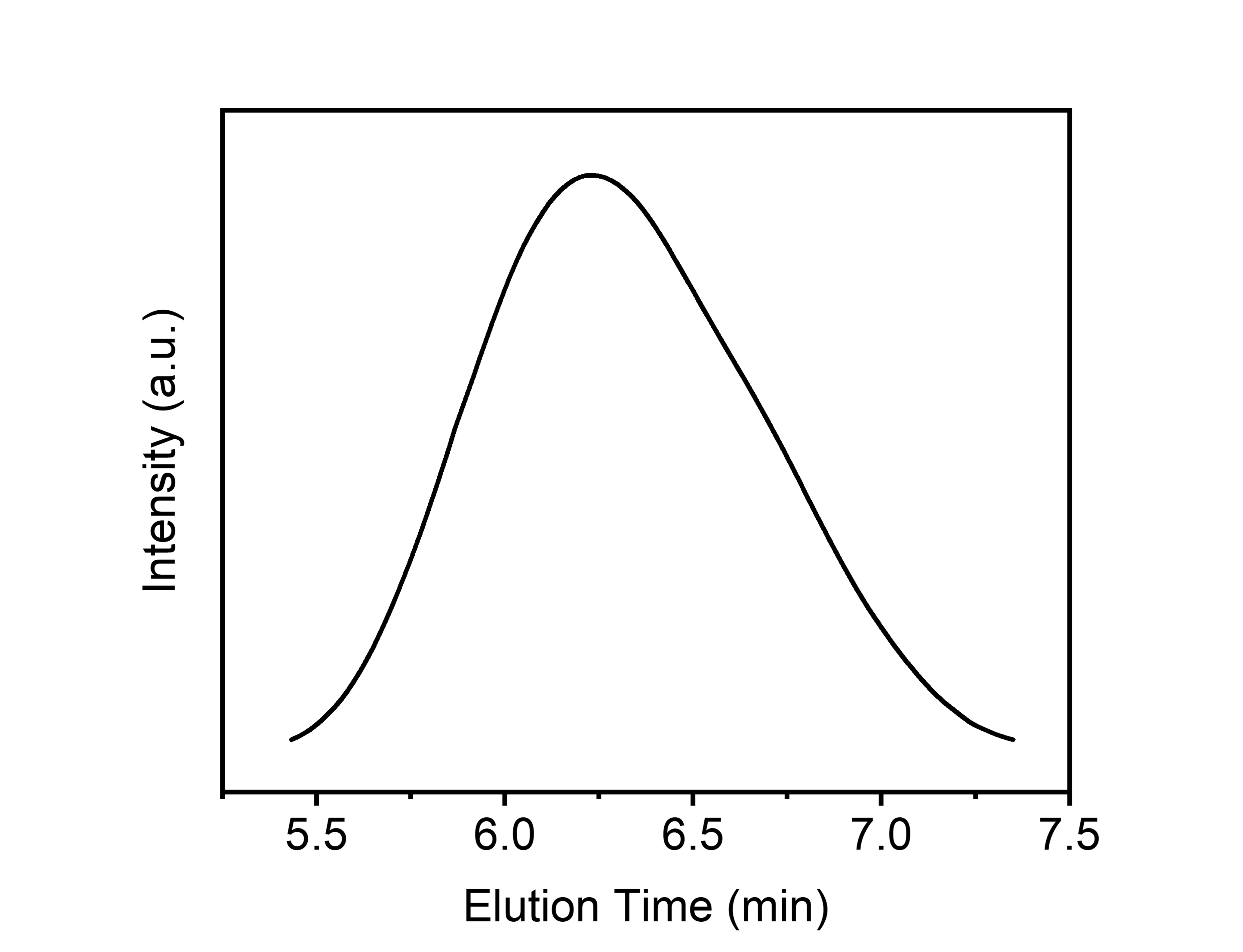


**Figure S2. Gel permeation chromatography (GPC) trace of PU.**


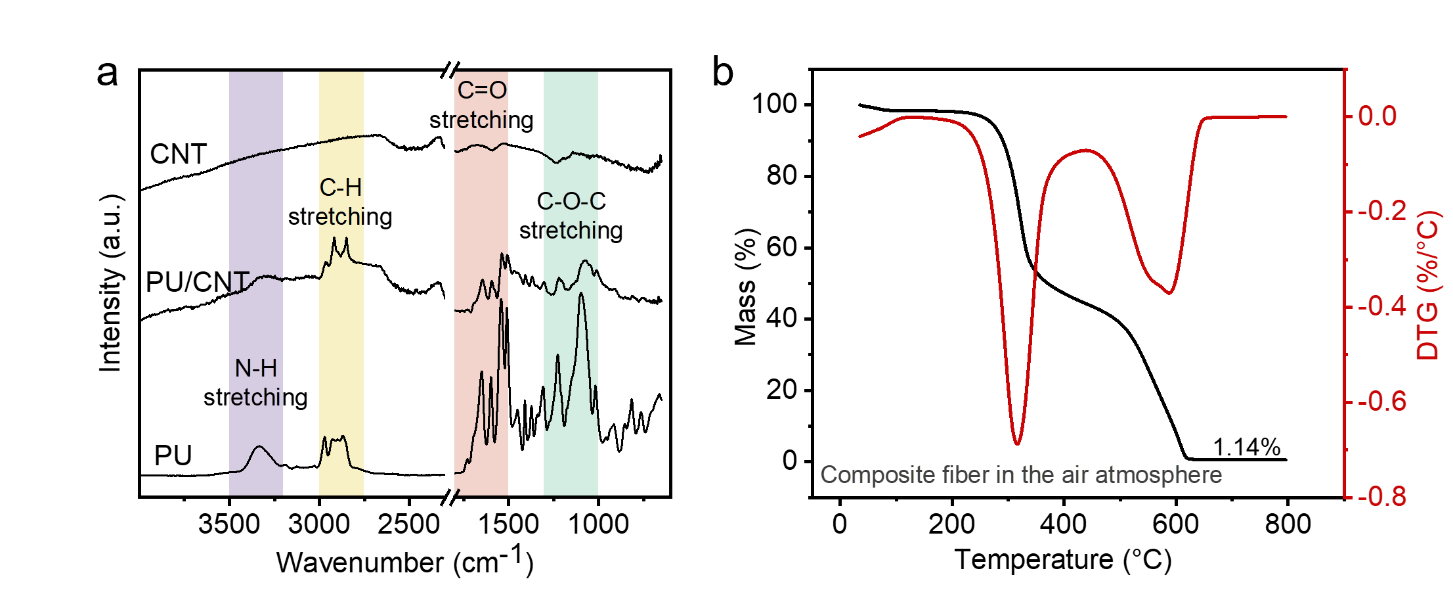


**Figure S3. Additional characterization of PU/CNT16.6 fibers.** (a) Fourier Transform Infrared Spectroscopy (FTIR) of PU/CNT16.6 fiber. (b) TG curve and DTG curve of the PU/CNT16.6 fiber in the air atmosphere.


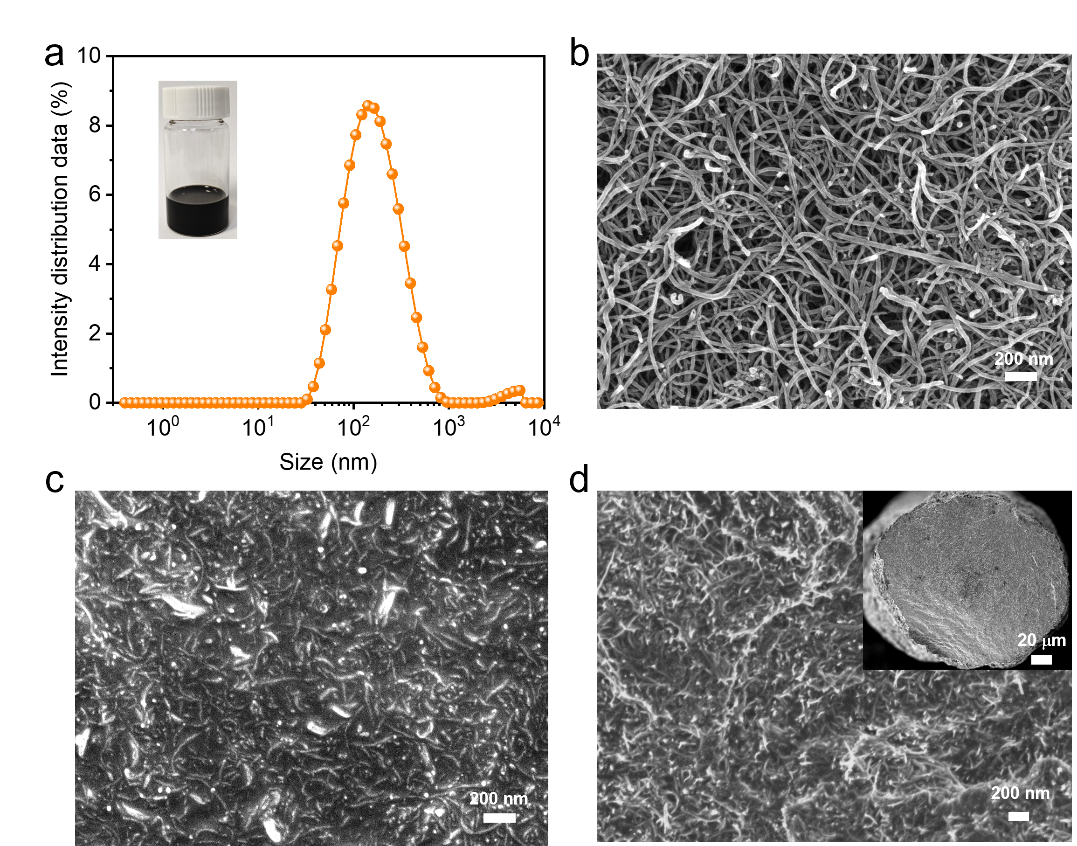


**Figure S4.** **Dispersion of CNTs in spinning liquids and PU/CNT fiber.** (a) Particle size distribution of the pre-dispersed CNTs solution. The primary particles in the solution are concentrated around 100 nm. The inset shows an optical photograph of the pre-dispersed solution, where the CNTs are uniformly and stably distributed in DMF. (b) SEM image of the pre-dispersed solution. No CNTs agglomeration is observed in the pre-dispersed solution after direct solvent drying. (c) SEM image of the spinning solution. After solvent drying, the CNTs are well dispersed and interconnected within the PU matrix. (d) SEM image of the PU/CNT16.6 fiber cross-section. The inset shows a macroscopic view of the fiber cross-section.


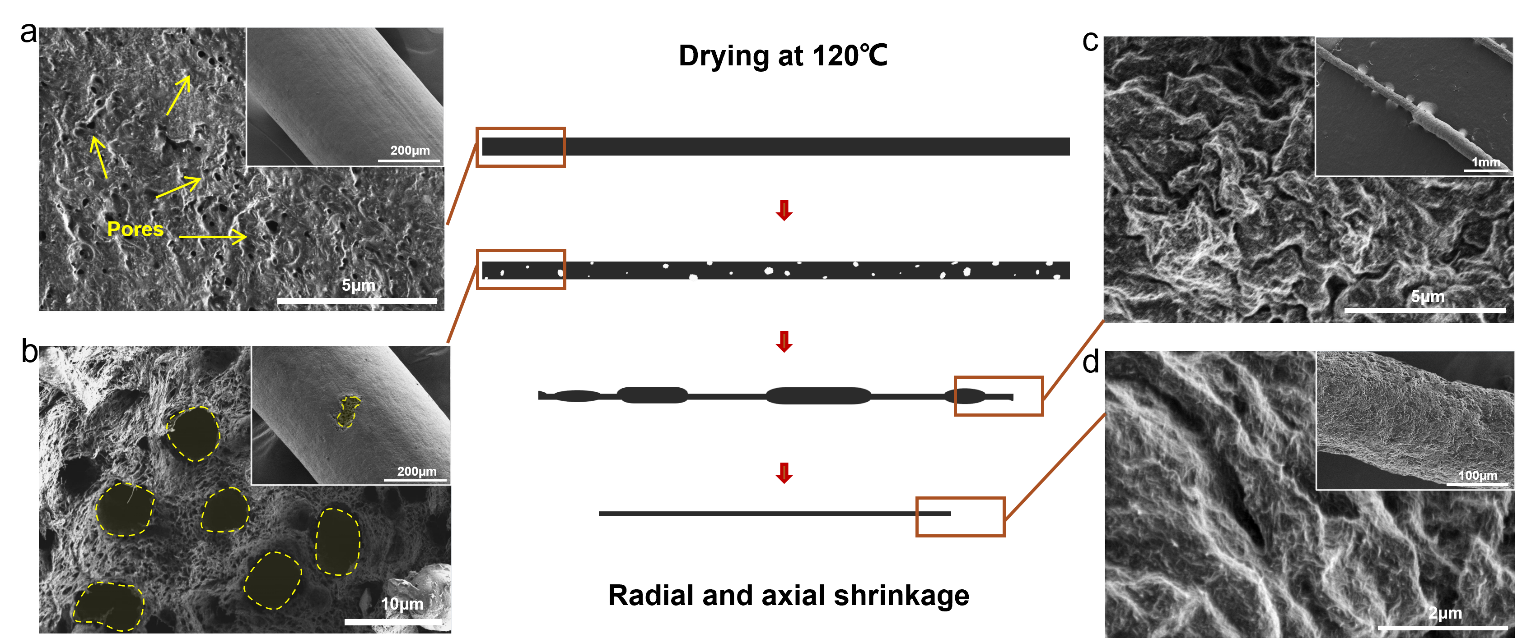


**Figure S5. SEM** **images of the PU/CNT fiber during the drying process.** The schematic in the middle simply shows the shrinkage of the PU/CNT and the gradual disappearance of the cavities under thermal treatment. (a) - (b) SEM images of PU/CNT before the drying process. A large number of cavities exist on the surface and in the interior of the fiber due to solution exchange in the solidification bath. (c) - (d) SEM images of fibers during and after the drying process. The fiber contracts and its surface roughens during heat treatment (120℃). Insets are SEM images with smaller magnification.


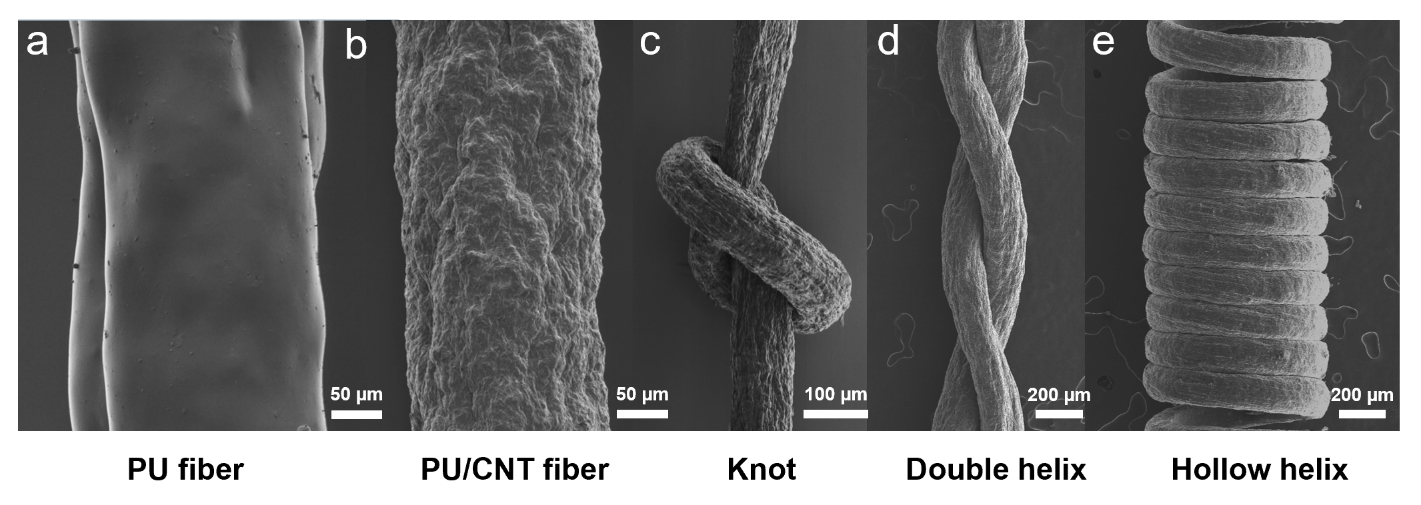


**Figure S6. Additional SEM images.** (a) Neat PU fibers obtained by wet spinning. (b) Thermally densified PU/CNT composite fiber with rough surface. (c) SEM images of a flexible fiber that can be knotted. (d) Double helix made by twisting two PU/CNT fibers. (e) Hollow helix made by PU/CNT.


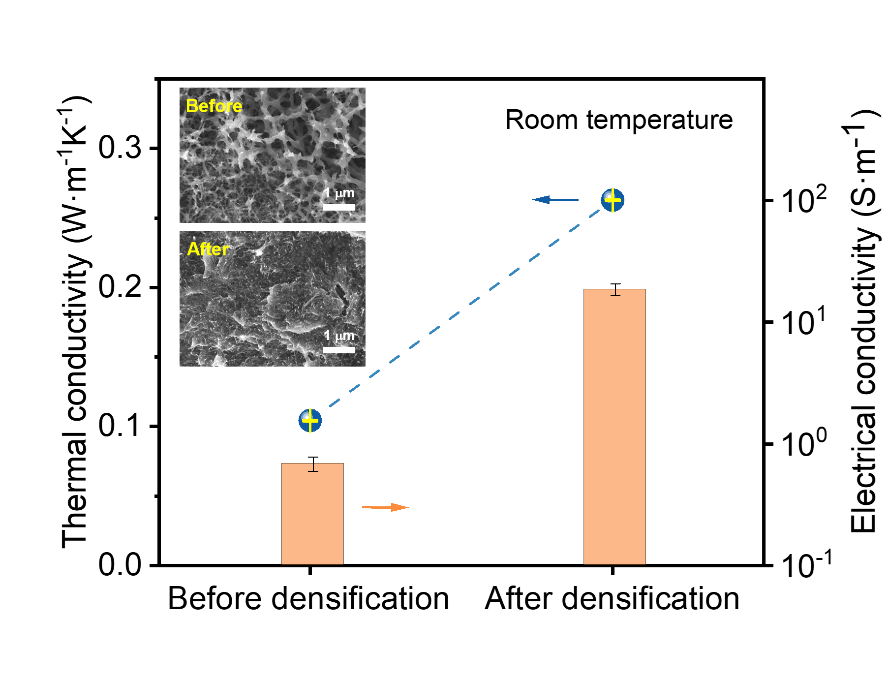


**Figure S7.** **Changes in thermal and electrical conductivity of the PU/CNT fiber before and after densification.** The inset shows cross-sectional SEM images of the fibers before and after densification, clearly displaying the changes in fiber porosity.


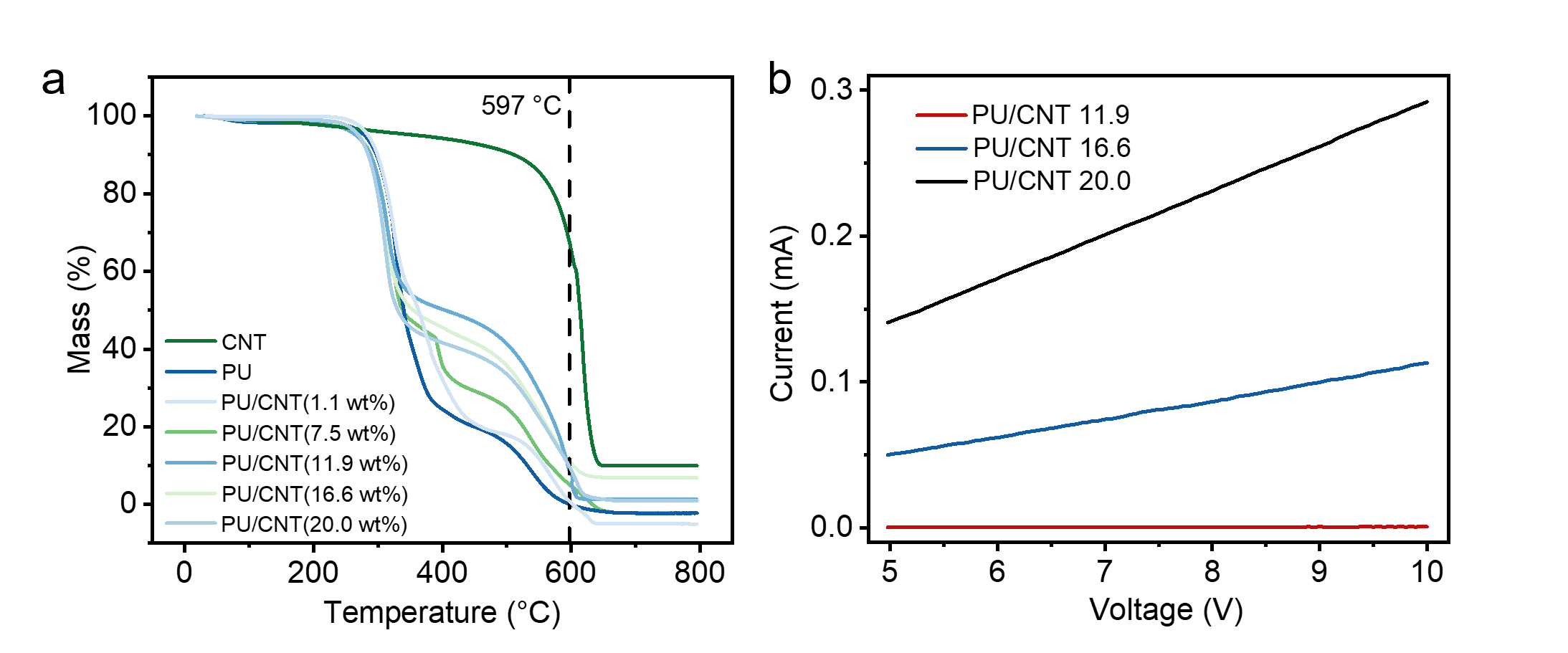


**Figure S8. Characterization of composite fibers with different CNT loadings.** (a) TG curves obtained in air for pristine CNT, neat PU and PU/CNT fibers with different CNT loadings. According to the data from the TG curves, the specific content of CNTs in each composite fiber can be calculated as 1.1 wt%, 7.5 wt%, 11.9 wt%, 16.6 wt% and 20.0 wt%, respectively. (b) Plot of current versus voltage curves of PU/CNT fibers in electrical measurement. The composite fibers have a conductive network with increasing CNT loading (at or above 11.9 wt%).


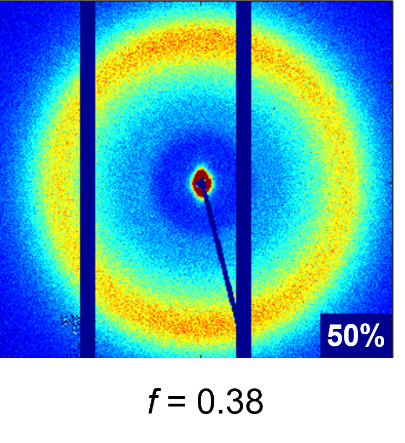


**Figure S9.** **2D-WAXS plots of a neat PU fiber stretched to 50% strains.** The pattern shows a clear splitting phenomenon with a Hermann orientation factor of *f*=0.38.

**
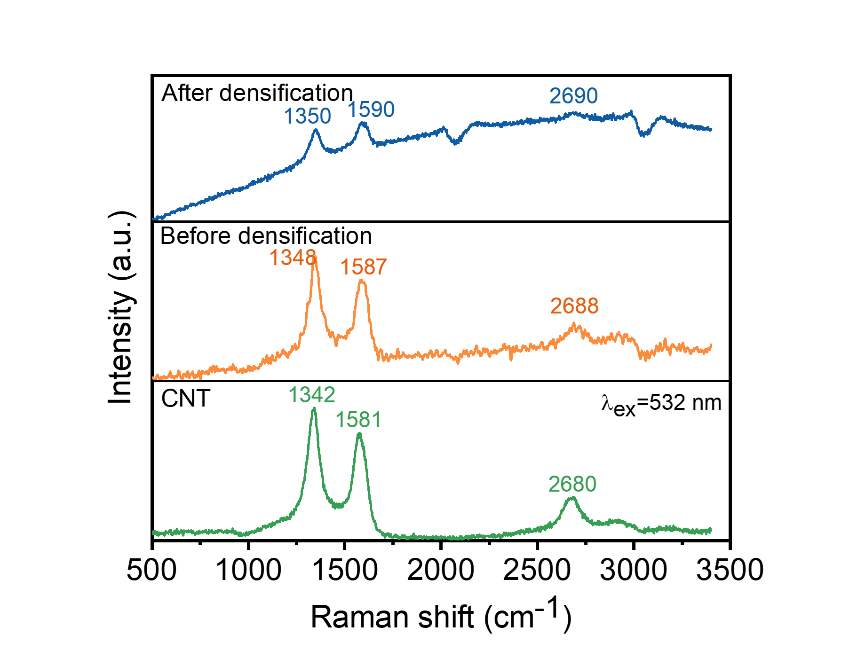
**

**Figure S10.** **Raman spectra of CNTs and composite fibers before and after densification.** The spectra show two typical carbon material features, the D band around 1350 cm^-1^ and the G band around 1580 cm^-1^. Compared to fibers without densification, the D and G bands of CNTs in the densified composite fibers upshifted (by 2 cm^-1^ and 3 cm^-1^, respectively).


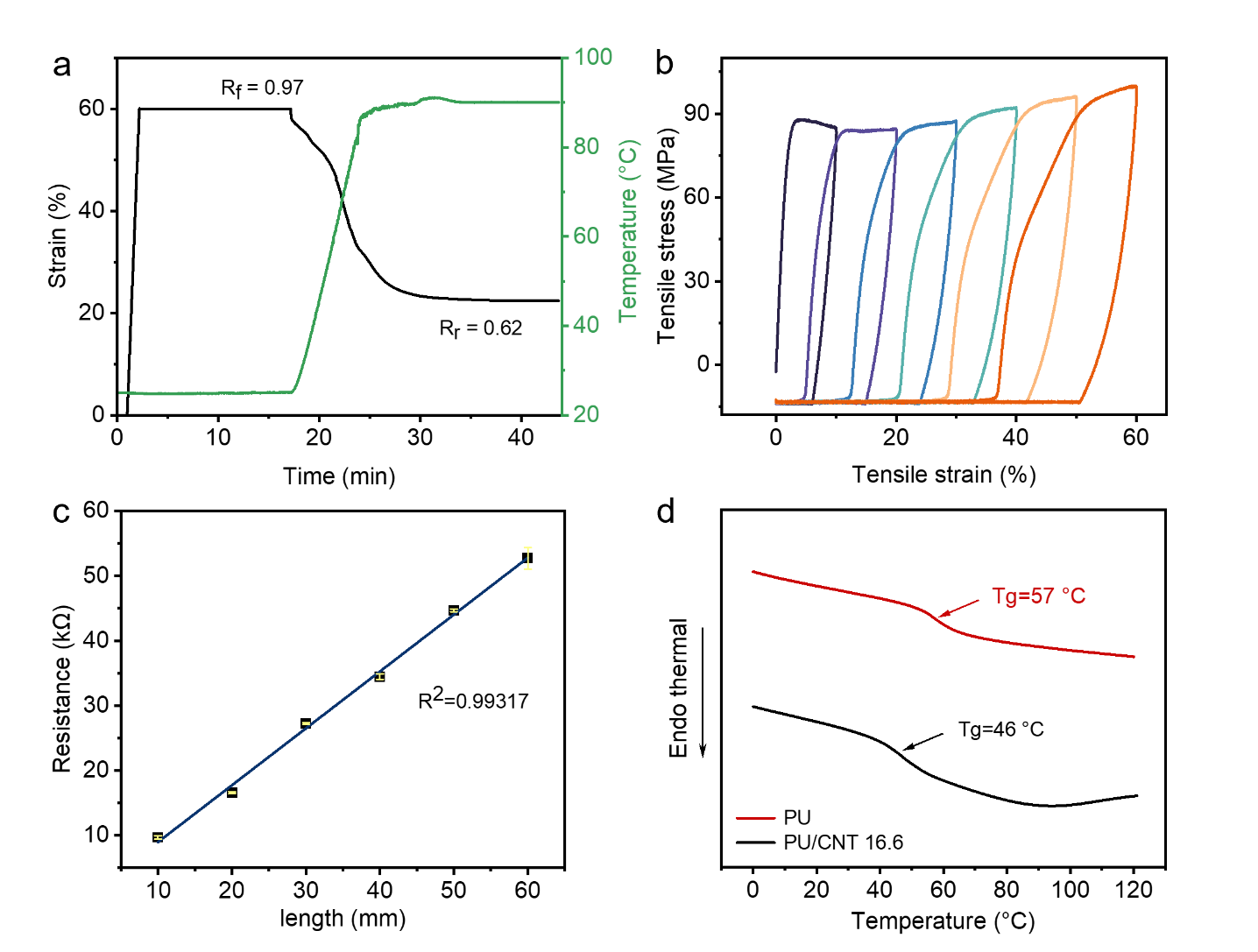


**Figure S11.** **Characterization of additional properties of the PU/CNT 16.6 fiber.** (a) Strain Recovery Experiment for PU/CNT 16.6 at 60% pre-strain. (b) Cycling stress-strain curves of PU/CNT 16.6 at room temperature with no reduction in fiber stress over cycles. (c) Curve of the resistance of PU/CNT 16.6 versus its length. The composite fiber resistance increases linearly with length. (d) DSC heating curves of PU and PU/CNT 16.6. The glass transition temperature (T_g_) of neat PU is 57 °C. The T_g_ of the composite fiber was reduced to 46 °C with a CNT loading of 16.6 wt%.


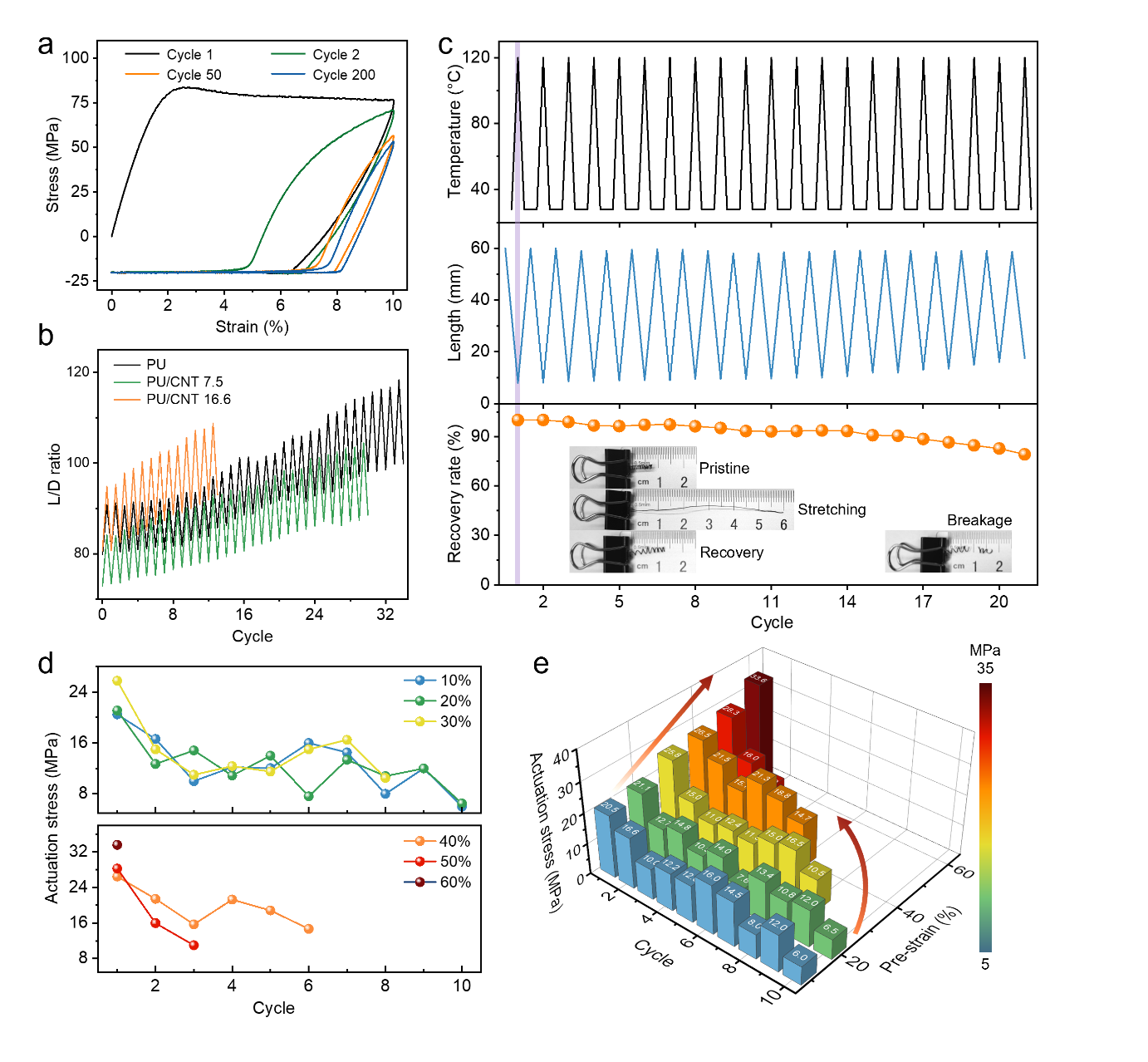


**Figure S12. Cyclic actuation properties of PU/CNT fibers.** (a) Stress-strain curve of the fiber under 10% strain for 200 cycles. It exhibited relaxation and gradually increasing residual strain during repeated tensile cycling. After 200 cycles, the stress decreased by approximately 30%. (b) Change in aspect ratio during thermal recovery cycling (under 10% strain) of composite fibers with different CNT contents (0%, 7.5% and 16.6%). The pure PU fiber broke after 34 cycles, the PU/CNT7.5 fibers lasted 30 cycles, and PU/CNT16.6 fibers only lasted 13 cycles. (c) Dimensional changes and corresponding recovery rate of the composite fiber in a spring shape during repeated actuation. The fiber pre-programmed into a hollow spring was straightened and fixed at room temperature. When heated to 120°C, the spring shape could recover and broke during the 21st stretching cycle. (d)-(e) Changes in actuation stress during thermal cycling of composite fiber (PU/CNT16.6). Pre-strain levels ranged from 10% to 60%.


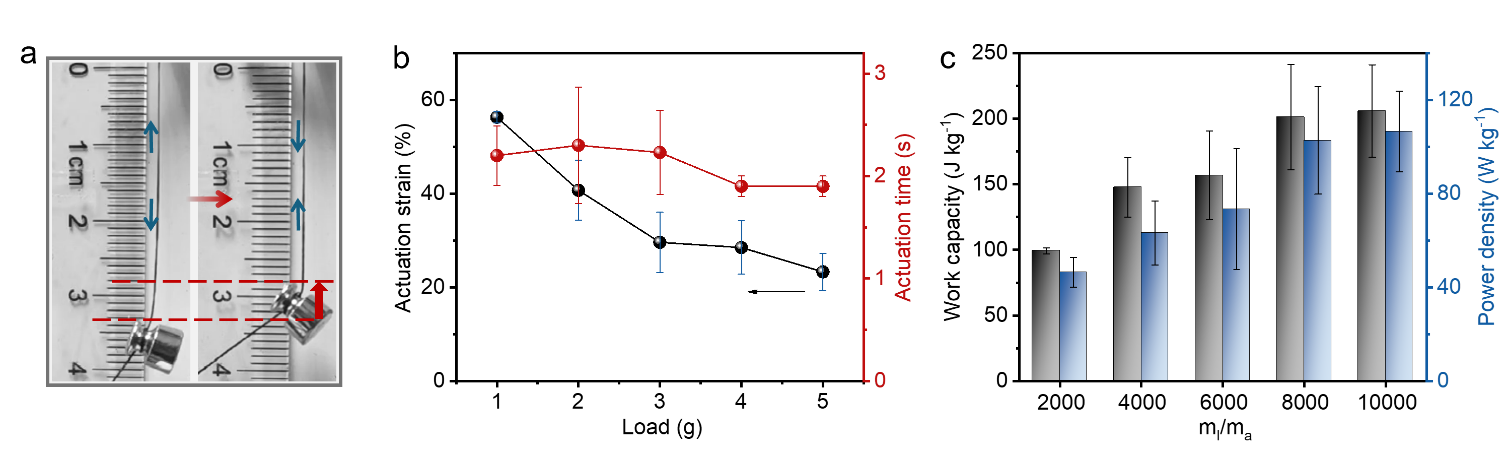


**Figure S13. Demonstration of weight lifting by PU/CNT Fibers under thermal actuation.** (a) Photos of PU/CNT fiber lifting a weight of 2 g by thermal actuation. Heat source is provided by a hot air gun. (b) Measured actuation strains and time for lifting different loads (1-5 g) by PU/CNT fibers. All lifting actuations were completed within 3 seconds, with a maximum actuation strain of 56% observed under a load of 1 g. (c) Calculated work capacities and power densities of PU/CNT fibers. Maximum work capacity (206 J kg^-1^) was obtained at a load-to-mass ratio of 10,000, along with a maximum energy density of 106 W kg^-1^. (The error bars are standard deviations calculated from three sets of data in this paper.)

The actuation strain (ε) was calculated using the following formula:

$$\varepsilon=\frac{L_{1}-L_{0}}{L_{1}-L_{2}} \left( 1 \right)$$

where $L_{0}$ is the original length of the fiber, $L_{1}$is the length of the pre-stretched fiber, and $L_{2}$ is the length of the fiber after recovery.

The work capacity (W) and power density (P) were calculated according to the following formula.

$$W=\frac{m_{l}}{m_{a}}gh \left( 2 \right)$$

$$P=\frac{W}{t} \left( 3 \right)$$

where $m_{l}$ is the load mass, $m_{a}$ is the actuator mass, $h$ is the distance the load is lifted, $g$ is the acceleration of gravity (9.8 m s^-2^), and $t$ is the time of lifting the load.


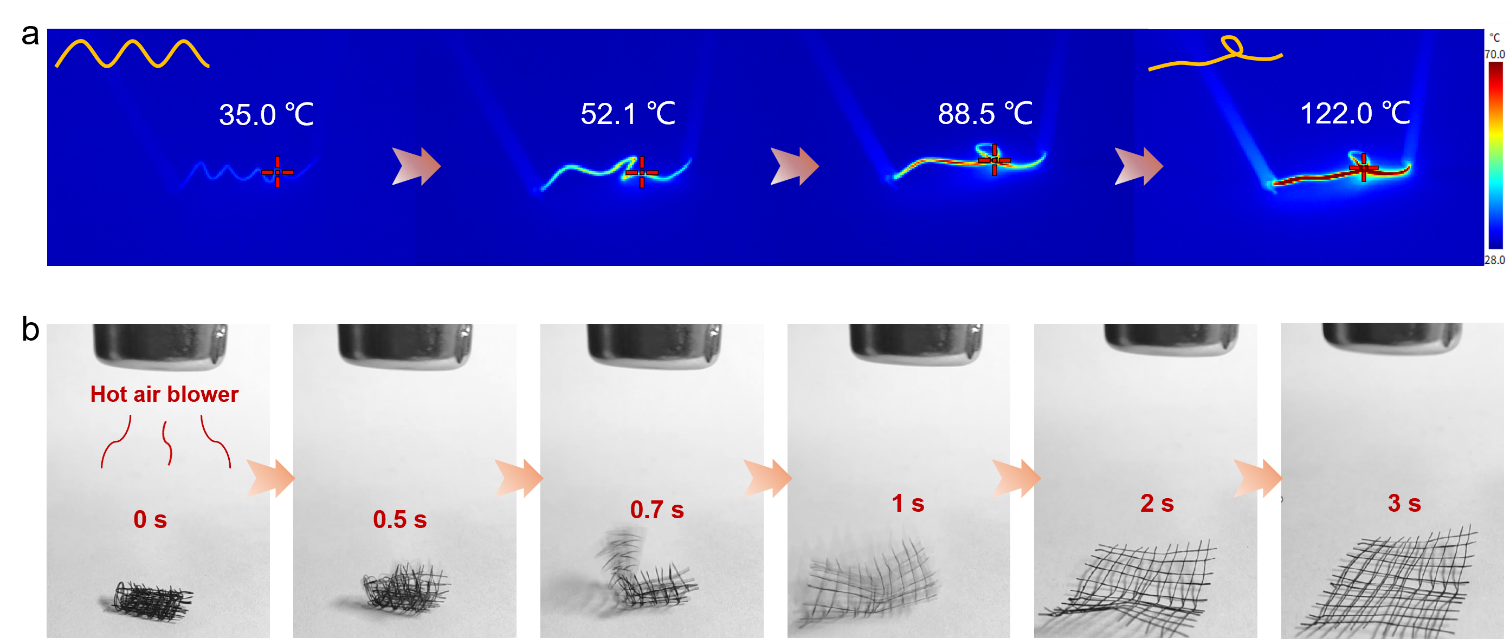


**Figure S14. Additional demonstration of pre-patterned PU/CNT fibers and their shape recovery process.** (a) Infrared images of a curved shape fiber and its electrical actuation recovery (the applied is 2 mA). The curved shape is recovered and curled in a limited space due to the electrode clamp fixation at both ends. (b) Heat gun provides uniform heating in the space. A folded mesh structure by interconnecting multiple PU/CNT fibers can be quickly unfolded into its original flat shape within 3 s.

**Table S1. Comparison of the actuation stresses among different composite actuators reported in literature.**

| **Materials** | **Actuation stress (MPa)** | **Actuation strain (%)** | **Stimulus** | **Ref.** |
| --- | --- | --- | --- | --- |
| Graphene oxide/SPU | 0.78 | 100 | Heat/ Electricity | 1 |
| CNT-PEDOT:PSS | 1 | 68 | Water | 2 |
| COCe-PE fiber | 5 | 47.7 | Heat | 3 |
| LCE/CNT fiber | 17.7 | 56.9 | Electricity | 4 |
| CNT/LCE fiber | 1.97 | 12 | light | 5 |
| MWNT yarns | 17.8 | 1.3 | Electricity | 6 |
| PW/CNT yarn | 14.3 | 3 | Electricity | 7 |
| SMPU-DBA | 2.02 | 20 | Heat | 8 |
| EPDM/lignin | 1.5 | 40 | Heat | 9 |
| CNT/Nylon-6 | 1 | 49 | Heat/ Electricity | 10 |
|  | 8.4 | 12 |  |  |
| CNT/Nylon-6,6 | 22 | 21 |  |  |
|  | 50 | 9.3 |  |  |
| Nylon 6,6 silver-plated | 22 | 10 |  |  |
| LCE microfiber | 0.3 | 60 | Heat/ light | 11 |
| Graphene/LCE | 0.24 | 2 | light | 12 |
| Graphene LCE | 1.23 | 47 | light | 13 |
| Hydrogel/CNT | 0.6 | 95 | Water | 14 |
| LCE/CNT/CNCs | 1.02 | 15 | Light/ Electricity | 15 |
| PEO-SO_3_@CNT | 33 | 16 | Water /Electricity | 16 |
| CNT yarn/Epoxy | 6.8 | 12 | Electricity | 17 |
| Nature melanin/PVA-co-PE/PA6 | 1 | 6.36 | light | 18 |
| LCE-MWCNTs/AgNWs | 0.46 | 39 | Electricity | 19 |
| Nanostructured block copolymer | 5.5 | 80 | Heat/Water | 20 |
| PU/CNT | 33/22 | 56.3/37.5 | Heat/ Electricity | This work |

**Supplementary Movie S1-S3.**

Movie S1 shows the lifting capacity of PU/CNT (effective length of 15 mm, and 60% pre-strain) composite fibers. (under both thermal/electrical actuation)

Movie S2 demonstrates the deformation of PU/CNT composite fibers with preprogrammed 2D shapes under both thermal and electrical actuation.

Movie S3 exhibits spatial deformation of PU/CNT composite fibers with 3D shapes.

[1] a) T. Ellringmann, C. Wilms, M. Warnecke, G. Seide, T. Gries, *Text. Res. J.* **2016**, 86, 178; b) S. Nunna, P. Blanchard, D. Buckmaster, S. Davis, M. Naebe, *Heliyon* **2019**, 5, e02698.

[2] a) L. Sun, Y. Zhang, *Toxicol Ind Health* **2018**, 25, 742; b) M. Kim, D. Goerzen, P. V. Jena, E. Zeng, M. Pasquali, R. A. Meidl, D. A. Heller, *Nat. Rev. Mater.* **2024**, 9, 63.
